# Perceived attitudes toward LGBTQ + physicians among individuals with psychiatric illness in the religiously diverse Southern Region of Thailand: a multihospital-based survey

**DOI:** 10.1186/s40359-024-01666-0

**Published:** 2024-03-25

**Authors:** Jarurin Pitanupong, Garn Wichitnark, Pakawat Wiwattanaworaset, Laddaporn Tepsuan, Naruttha Norphun, Puangsan Yakkaphan

**Affiliations:** 1 Department of Psychiatry, Faculty of Medicine, Prince of Songkla University, Hat Yai, Songkhla, 90110 Thailand; 2 Songkhla Hospital, Mueang Songkhla District, Songkhla, 90000 Thailand; 3 Songkhla Rajanagarindra Psychiatric Hospital, Mueang Songkhla District, Songkhla, 90000 Thailand; 4 Pattani Hospital, Mueang Pattani District, Pattani, 94000 Thailand

**Keywords:** Attitude, LGBTQ +, Patient, Physician, Psychiatric illness

## Abstract

**Objectives:**

This study purposed to analyze perceived attitudes toward LGBTQ + physicians and related factors among individuals with psychiatric illnesses in southern Thailand.

**Materials and methods:**

From May to July 2023, a cross-sectional study was conducted at four psychiatric outpatient clinics in Southern Thailand. The questionnaires utilized were: 1) a demographic information questionnaire, 2) a questionnaire regarding attitudes toward LGBTQ + physicians, 3) a questionnaire evaluating individuals’ attitudes toward LGBTQ + physicians while receiving medical attention, and 4) a patient-doctor relationship questionnaire. All data were analyzed using descriptive statistics, and the factors associated with perceived attitudes toward LGBTQ + physicians were analyzed using the Wilcoxon rank sum test, the Kruskal–Wallis test, and linear regressions. The analyses were conducted using the R Foundation for Statistical Computing software, version 4.3.1. Statistical significance was defined as a *p*-value of less than 0.05.

**Results:**

Among our 542 participants, the mean age was 36.3 ± 14.1 years. The majority were female (64.6%), Buddhist (62.4%), and diagnosed with depression (46.3%). Approximately three-quarters showed a good doctor-patient relationship (74.0%). The median (IQR) score of the perceived attitudes toward LGBTQ + physicians was 75 (66, 88). Predominantly, the LGBTQ + physicians were perceived as normal (76.3%) and being a viable part of society (88.7%). Moreover, our participants disagreed with the view that being an LGBTQ + physician was a sin (70.6%) or immoral (68.2%). They felt comfortable during history taking (79.0%), physical examination not involving private parts of the body (72.5%), and management for both medical (78.4%) and psychiatric conditions (81.4%) at the hands of LGBTQ + physicians. However, they reported feeling uncomfortable during history taking involving private matters (6.3%) and the physical examination of private parts (16.4%). Older age, absence of LGBTQ + close relatives/friends, and being a Muslim were associated with lower scores of perceived attitudes toward LGBTQ + physicians. Conversely, a higher level of education and a reported mismatch between the patient’s sex and gender were associated with higher scores.

**Conclusions:**

Most participants reported positive perceived attitudes toward LGBTQ + physicians. However, some age groups and adherents of Islam showed lower perceived attitude scores and reported feeling uncomfortable receiving medical treatment from LGBTQ + physicians. On the one hand, LGBTQ + physicians have cause to be concerned about this point; on the other hand, finding appropriate approaches to promote positive attitudes toward LGBTQ + physicians among these groups of people remains a necessity.

## Introduction

Lesbian, gay, bisexual, transgender, queer or questioning, and more (LGBTQ +) individuals represent sexual and gender minorities, who face substantial stigma [1, 2]. Within a culture that reinforces binary gender classifications or roles, normalizes heterosexuality, and often considers departing from these norms as a pathology, LGBTQ + individuals may easily develop sexual stigma [3].

Regarding the patient-physician relationship, a 2003 study identified some barriers to providing optimal care that existed between physicians and LGBTQ + adolescents. The majority of physicians were not confident in themselves in all the skills to address issues of gender identity, sexual orientation, or sexual attraction while taking their sexual history with LGBTQ + adolescent patients. Moreover, the physicians suggested that these sexual issues should be addressed during their course of training [4].

The perception of the physician's identity, which included an attitude toward the mental image of the physician, the behavior and working experience of the physician, and the effect of media, or even advertisements on creating an image of the physician, had been identified as one of the main categories of barriers between the patient and the physician [5]. Prior studies have reported that it is not only physicians who have prejudiced feelings or sexual stigma toward LGBTQ + patients but that patients might also be prejudiced against LGBTQ + physicians [6, 7]. However, prior studies on LGBTQ + topics in the medical field have always focused on surveying the attitude of physicians or healthcare workers toward LGBTQ + people [8]. There are very few studies on people's or patients' attitudes toward LGBTQ + physicians. In Thailand, only two prior studies have been conducted in the Southern Region about patients’ attitudes toward overtly effeminate male or overtly masculine female physicians. In 2009, the first study identified that almost all patients thought that overtly effeminate male physicians or overtly masculine female physicians were acceptable [9]. Only 2.4% and 1.4% of patients reported that they felt distressed and nervous to have contact with them [9]. In 2010, the second study identified that most southern Thai patients and their relatives had a positive attitude toward overtly effeminate male physicians or overtly masculine female physicians [10]. However, patients and their relatives who were male, adhered to Islam religions, had a lower level of education, were elderly (more than 60 years old) [9, 10], and belonged to the age group of 12–19 years old [9] were found to have some prejudiced attitudes towards overtly effeminate male physicians and/or overtly masculine female physicians [9, 10].

Nowadays, the attitudes toward LGBTQ + individuals may be more positive and widely spread. As far as Thai society is concerned, people’s attitudes regarding these issues have changed for the better in comparison to the past. Yet, the attitudes toward LGBTQ + physicians and their associating factors among individuals with psychiatric illnesses, who are a vulnerable population, have not, to our knowledge, been studied before in the Thai context. Because individuals with psychiatric illnesses may have a deficit in fulfilling relationships with others or have problems with mistrust of others [11]. Limited data exist concerning these issues in Thailand in general and in the Southern Region in particular. While the majority of Thailand’s population is Buddhist, most provinces in the Southern Region are religiously diverse, and some are predominantly Muslim. It can, therefore, be inferred that the Southern peoples’ values and beliefs may vary from those of populations from the other Thai regions where the demographic characteristics in terms of religious affiliation are not the same. Hence, this study purposed to analyze the perceived attitudes toward LGBTQ + physicians and their related factors among Thai individuals with psychiatric illnesses attending psychiatric outpatient clinics at four hospitals in Southern Thailand, which serve large numbers of patients with diverse religious affiliations. In addition, since the doctor-patient relationship may be affected by the patient’s attitudes, it was used as an associating factor in the data analyses. We believe that our findings may provide valuable knowledge toward the establishment of appropriate encounter settings that promote a good relationship among LGBTQ + physicians and Thai individuals with psychiatric illnesses as well as an excellent care process.

In this research, the participant LGBTQ + physicians were defined as physicians whose gender identity or gender expression was not congruent with their biological sex.

## Materials and methods

From May to July 2023, after approval from the corresponding ethics committees of the Faculty of Medicine, Prince of Songkla University (REC: 65–474-3–4), Songkhla Hospital (SKH IRB 2023-Md-J3-1043), Pattani Hospital (PTN-007–2566), and Songkhla Rajanagarindra Psychiatric Hospital (SKPH.IRB 1/2566), this cross-sectional study was conducted using questionnaires at four psychiatric outpatient clinics belonging to Songklanagarind Hospital, which is an 800-bed university hospital serving as a tertiary center; Songkhla Hospital and Pattani Hospital, which are both 508-bed general provincial hospitals; and Songkhla Rajanagarindra Psychiatric Hospital, which is a 200-bed psychiatric hospital serving as a referral center in Southern Thailand. These four hospitals serve a large number of patients from diverse religious affiliations at their psychiatric outpatient clinics; this ensured the religious diversity of our study population.

The study sample comprised all the individuals with a psychiatric illness, who attended the four above-mentioned psychiatric outpatient clinics, were at least 20 years of age, could understand and use the Thai language for verbal communication, and could read and complete the questionnaires. Psychiatric illness included depression, anxiety, psychosis, and substance use in which symptoms were in remission. The patients who lacked the mental capacity to complete all of the questionnaires, such as those with severe psychotic symptoms, severe depression or mania, severe intoxication, or withdrawal features, were excluded. Their mental capacity had been screened and judged by an outpatient psychiatric nurse.

In terms of sample size calculation, a prior study reported that 14.9% of its patient population considered being an overtly effeminate male or an overtly masculine female doctor unacceptable in Thai society [10], so this study used P as equal to 14.9% to calculate the sample size from the following formula.

$$n=\frac{Z_\frac a2^2PQ}{d^2}$$

d = 0.03 (approximately 20.0% of p), Zα/2 represented the critical value of the normal distribution at α/2 (e.g., for a confidence level of 95.0%, α was 0.05, and the critical value was 1.96), d was the margin of error, and p was the proportion of patients who acknowledged that being an overtly effeminate male or an overtly masculine female doctor was unacceptable in the Thai society. Hence, it was determined that the study needed at least 541 patients. We then divided them into equal proportions to ensure a fair representation of the demographic characteristics of each hospital. The sample size per hospital was 136 patients.

The data collection was performed following a convenience process and the relevant guidelines by using a paper-based process. After the outpatient psychiatric nurse had assessed the mental capacity of all the individuals with a psychiatric illness who attended the specified psychiatry outpatient clinics and fit the inclusion criteria, our research assistants approached all of them. An information sheet, which delineated the rationale for the study and the allotted time to complete the survey, was distributed to them. They were given 20–30 min to consider whether to participate in the study or not. If they agreed to participate, the research assistant would hand them the questionnaires and an informed consent was signed by the patients. The researcher assistant would then invite them to a private space to conduct the survey, observed the participants’ responses, and advised them if they felt distressed or uncomfortable. The participants completed the questionnaire independently, without additional guidance from the questionnaire distributor. Furthermore, it was made clear to the patients that they could terminate the interview and withdraw from the research at any time without any concern for repercussions as to their medical treatment or otherwise.

## Questionnaires

1. The general demographic information sheet inquired around areas associated with age, gender, sex, religion, marital status, education, income, occupation, psychiatric diagnosis (by the owner psychiatrist and being retrieved from the medical register), presence of LGBTQ + close relatives/friends, and experience with LGBTQ + acquaintances and physicians.
2. The Attitudes Toward LGBTQ + physician questionnaire is a self-rating questionnaire that was adapted from the Attitudes Toward Transgendered Individuals Scale (ATTI) Thai version. As the ATTI questionnaire consisted of twenty questions aimed at assessing cognitive evaluations and affective reactions to transgendered individuals and transgenderism. The ATTI scale comprised general and universal questions that could be applied to LGBTQ + people, the wording “transgendered individual/transgenderism” was replaced with “LGBTQ + physician/being an LGBTQ + physician.” The Attitudes Toward LGBTQ + Physicians questionnaire underwent content validity assessment by five psychiatrists. The resulting content validity index (CVI) score was 0.8. Concerning to twenty questions, the scoring of each question employed a 5-point Likert-type response: 1 (strongly agree); 2 (agree); 3 (neither agree nor disagree); 4 (disagree); 5 (strongly disagree). Moreover, nine items (1, 5, 8, 10, 12, 13, 14, 16, and 17) were reverse-scored, and the scores of all twenty items were summed to create a total score with a potential range between 20 and 100. Higher scores reflected more positive attitudes toward LGBTQ + physicians [12, 13]. This questionnaire has been found to have a high internal consistency and reliability, indicated by a Cronbach’s alpha coefficient of 0.96 [13]. Meanwhile, The Attitudes Toward LGBTQ + Physicians questionnaire demonstrated good internal consistency for the data of this study as evidenced by a Cronbach's alpha coefficient of 0.94.
3. The self-rating questionnaire compared the individuals’ attitudes toward LGBTQ + physicians with those toward non-LGBTQ + physicians during the six stages of the care process they received at the hospital: taking a history of general matters; taking a history of private matters such as sexual relationships and psychiatric illness; physical examination involving the general body parts; physical examination of private body parts such as breasts and genitalia; management for physical medical conditions; and management for psychiatric conditions. The score of each question ranged from 1 to 3; 1 (uncomfortable); 2 (neutral); and 3 (comfortable) [9, 10, 14].
4. The Patient-Doctor Relationship Questionnaire (PDRQ-9) Thai version is a self-rating questionnaire that was adapted from its original English version, which has a content validity index (CVI) score of 0.8 [15]. It comprised nine questions and employed a 5-point rating scale: 1 (not at all appropriate); 2 (somewhat appropriate); 3 (appropriate); 4 (mostly appropriate); and 5 (totally appropriate). The higher score reflected a good patient-doctor relationship. The total score was summed up and ranged from 9 to 45; their interpretation is as follows: 36 or higher (good doctor-patient relationship); 18 to 35 (moderately good doctor-patient relationship); and 17 or lower (poor doctor-patient relationship) [16]. The original questionnaire was reported to have a Cronbach's Alpha coefficient of 0.7–0.94 [17, 18]. The PDRQ-9 questionnaire demonstrated good internal consistency for the data set of this study on account of Cronbach's alpha coefficient of 0.95.

## Statistical analysis

Descriptive statistics, such as proportion, mean, standard deviation (SD), median, and interquartile range (IQR), were calculated. The difference between demographic characteristics (such as age, religion, level of education, marital status, etc.) and perceived attitude towards LGBTQ + physicians in univariate analysis was performed using the Wilcoxon rank-sum test and the Kruskal–Wallis test. Linear regression analyses were used to identify associations with perceived attitudes toward LGBTQ + physicians. The analyses were conducted using the R software of the Foundation for Statistical Computing, version 4.3.1. Statistical significance was defined as a *p*-value of less than 0.05.

## Results

### Demographic characteristics

Of all individuals with a psychiatric illness who attended the four psychiatric outpatient clinics from May to July 2023 and met the inclusion criteria, 544 agreed to participate in this study. However, 2 participants failed to complete the questionnaires in their entirety. The median (interquartile range [IQR]) age of our study population (*n* = 542) was 33 (25, 46) years. The majority of the participants were female (64.6%), Buddhist (62.4%), single (56.6%), and educated at a Bachelor's degree or higher level (48.2%). There were 21 participants (3.9%) who reported a sex-gender mismatch. In terms of psychiatric illnesses, major depressive disorder (MDD) (46.3%) and generalized anxiety disorder (GAD) (20.3%) were the most often reported among the participants. With respect to the patient-doctor relationship findings using the PDRQ-9 questionnaire, most participants (74.0%) reported a good doctor-patient relationship (Table 1).

**Table 1:** Demographic characteristics (*N* = 542)

Demographic characteristics	Number (%)
**Sex**	
Male	191 (35.2)
Female	350 (64.6)
No answer	1 (0.2)
**Gender**	
Male	185 (34.1)
Female	340 (62.7)
Other	16 (3.0)
No answer	1 (0.2)
**Matched sex and gender**	
Male	182 (33.6)
Female	338 (62.4)
Mismatch	21 (3.9)
No answer	1 (0.2)
**Marital status**	
Single	307 (56.6)
Married	197 (36.3)
Divorced/widowed/separated	35 (6.5)
No answer	3 (0.6)
**Religion**	
Buddhism	338 (62.4)
Islam	159 (29.3)
Christianity/other	45 (8.3)
**Level of education**	
Primary school or below	53 (9.8)
Secondary school	47 (8.7)
High school/diploma	177 (32.7)
Bachelor’s degree or above	261 (48.2)
No answer	4 (0.7)
**Occupation**	
Government officer/state enterprise employee/ company employee	139 (25.6)
Self-employed/merchant/personal business owner/ agriculture	216 (39.9)
Student	92 (17.0)
Unemployed	93 (17.2)
No answer	2 (0.4)
**Diagnosis**	
Depression	251 (46.3)
Anxiety/GAD	110 (20.3)
Panic disorder	39 (7.2)
Bipolar disorder	37 (6.8)
Schizophrenia	27 (5.0)
Substance use	20 (3.7)
Other	58 (10.7)
**Presence of LGBTQ + close relatives/friends**	
Yes	262 (48.3)
No	277 (51.1)
No answer	3 (0.6)
**Prior experience of medical examination by an LGBTQ + physician**	
Yes	196 (36.2)
No	342 (63.1)
No answer	4 (0.7)
**Patient-doctor relationship**	
Poor	5 (0.9)
Moderate	136 (25.1)
Good	401 (74.0)

### Attitudes toward LGBTQ + physicians

**Fig. 1:**
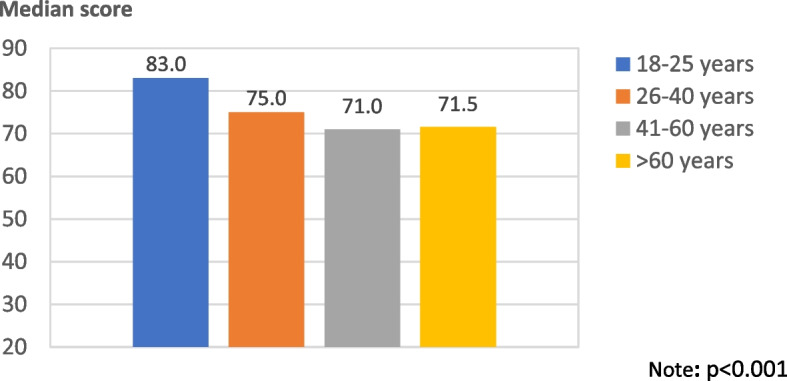
Average scores of perceived attitudes toward LGBTQ + physicians between age groups (*N* = 542)

Of all participants, 48.3% reported the presence of LGBTQ + close relatives/friends, and 36.2% had a previous experience being medically examined by LGBTQ + physicians (Table 1). Regarding attitudes toward LGBTQ + physicians, the median (IQR) score was 75 (66, 88). The average scores of the perceived attitude toward LGBTQ + physicians between age groups are shown in Fig. 1. There was a statistically significant difference between the average score of perceived attitude towards LGBTQ + physicians and age group (*p* < 0.001).

Most participants recognized LGBTQ + physicians as normal (76.3%) and a viable part of society (88.7%), and opined that they should be accepted completely into society (79.7%). In addition, they tended to disagree/strongly disagree with the view that being an LGBTQ + physician was a sin (70.6%) or immoral (68.2%) (Table 2).

**Table 2:** Attitudes toward LGBTQ + physicians (*N* = 542)

	**Strongly agree**	**Agree**	**Neither agree nor disagree**	**Disagree**	**Strongly disagree**
It would be beneficial to society to recognize LGBTQ + physicians as normal	198 (36.6)	215 (39.7)	101 (18.7)	18 (3.3)	9 (1.7)
LGBTQ + physicians should not be allowed to work with children	22 (4.1)	55 (10.2)	128 (23.7)	194 (35.9)	141 (26.1)
Being an LGBTQ + physician is immoral	9 (1.7)	47 (8.7)	116 (21.5)	172 (31.9)	196 (36.3)
All LGBTQ + bars should be closed down	19 (3.5)	72 (13.4)	147 (27.3)	166 (30.8)	135 (25.0)
LGBTQ + physicians are a viable part of our society	239 (44.3)	240 (44.4)	48 (8.9)	7 (1.3)	6 (1.1)
Being LGBTQ + is a sin	14 (2.6)	61 (11.3)	84 (15.5)	202 (37.3)	180 (33.3)
Being LGBTQ + endangers the institution of the family	9 (1.7)	30 (5.5)	91 (16.8)	217 (40.0)	195 (36.0)
LGBTQ + physicians should be accepted completely into our society	204 (37.7)	227 (42.0)	76 (14.0)	23 (4.3)	11 (2.0)
LGBTQ + physicians should be barred from the teaching profession	11 (2.0)	35 (6.5)	114 (21.0)	188 (34.7)	194 (35.8)
There should be no restrictions on being an LGBTQ + physician	144 (26.6)	170 (31.4)	133 (24.6)	73 (13.5)	21 (3.9)
I avoid LGBTQ + physicians whenever possible	9 (1.7)	35 (6.5)	121 (22.4)	197 (36.4)	179 (33.1)
I would feel comfortable working closely with an LGBTQ + physician	179 (33.1)	215 (39.7)	107 (19.8)	28 (5.2)	12 (2.2)
I would enjoy attending social functions where LGBTQ + physicians were present	157 (29.0)	176 (32.5)	175 (32.3)	27 (5.0)	7 (1.3)
I would feel comfortable if I learned that my neighbor was an LGBTQ + physician	173 (32.0)	200 (37.0)	134 (24.8)	28 (5.2)	5 (0.9)
LGBTQ + physicians should not be allowed to cross-dress in public	22 (4.1)	109 (20.1)	143 (26.4)	144 (26.6)	123 (22.7)
I would like to have friends who are LGBTQ + physicians	197 (36.6)	201 (37.4)	103 (19.1)	29 (5.4)	8 (1.5)
I would feel comfortable if I learned that my best friend was an LGBTQ + physician	203 (37.7)	208 (38.6)	94 (17.4)	27 (5.0)	7 (1.3)
I would feel uncomfortable if a close family member became romantically involved with an LGBTQ + physician	31 (5.7)	99 (18.3)	188 (34.7)	93 (17.2)	131 (24.2)
LGBTQ + physicians are just closeted gays	25 (4.6)	68 (12.6)	221 (41)	138 (25.6)	87 (16.1)
Romantic partners of LGBTQ + physicians should seek psychological treatment	21 (3.9)	42 (7.8)	136 (25.2)	180 (33.4)	160 (29.7)

When comparing the participants’ perceived attitudes toward LGBTQ + physicians with those toward non-LGBTQ + physicians while going through the six stages of the care process received at the hospital, we found that the participants reported feeling comfortable during taking a history of general matters (79.0%), physical examination of general body parts (72.5%), management for medical conditions (78.4%), and management for psychiatric conditions (81.4%). However, they reported feeling comfortable during history taking involving private matters (65.6%) and the physical examination of private body parts (48.3%) by LGBTQ + physicians in a lower proportion than that of the other care processes (Table 3).

**Table 3:** Attitudes toward LGBTQ + physicians related to the medical care processes (*N* = 542)

Medical care processes	Uncomfortable	Neutral	Comfortable
History taking covering general matters	11 (2.0)	103 (19.0)	428 (79.0)
History taking covering private matters such as sexual relationship and psychiatric illness	34 (6.3)	152 (28.1)	355 (65.6)
Physical examination of general body parts	24 (4.4)	125 (23.1)	392 (72.5)
Physical examination of private body parts such as breasts and genitalia	89 (16.4)	191 (35.2)	262 (48.3)
Management for medical conditions	14 (2.6)	103 (19.0)	424 (78.4)
Management for psychiatric conditions	15 (2.8)	86 (15.9)	441 (81.4)

In terms of the participants who reported feeling uncomfortable when receiving care from LGBTQ + physicians, being female, middle-aged, unmarried, Buddhist, educated at the Bachelor’s degree level or above, and the absence of LGBTQ + close relatives/friends as well as the lack of previous experience of being medically examined by an LGBTQ + physician were the major groups. In addition, the median scores of perceived attitudes toward LGBTQ + physicians of these groups were lower than that of the entire population (Table 4).

**Table 4:** Characteristics of participants who reported feeling uncomfortable during receiving the medical care processes by LGBTQ + physicians

	**Processes of medical management**					
	**Taking a history of general matters**	**Taking a history of private matters**	**Physical examination of general body parts**	**Physical examination of private body parts**	**Management for medical conditions**	**Management for psychiatric conditions**
**Number of participants** *N* (%)	11 (2.0)	34 (6.3)	24 (4.4)	89 (16.4)	14 (2.6)	15 (2.8)
**Matched sex and gender**						
Male	2 (18.2)	14 (41.2)	7 (29.2)	34 (38.2)	4 (28.6)	6 (40.0)
Female	9 (81.8)	20 (58.8)	17 (70.8)	54 (60.7)	10 (71.4)	9 (60.0)
Mismatch	0 (0.0)	0 (0.0)	0 (0.0)	1 (1.1)	0 (0.0)	0 (0.0)
**Age group (years)**						
18–25	2 (18.2)	8 (23.5)	5 (20.8)	20 (22.5)	2 (14.3)	2 (13.3)
26–40	0 (0.0)	10 (29.4)	11 (45.8)	35 (39.3)	5 (35.7)	4 (26.7)
41–60	6 (54.5)	10 (29.4)	7 (29.2)	26 (29.2)	6 (42.9)	7 (46.7)
> 60	3 (27.3)	6 (17.6)	1 (4.2)	8 (9.0)	1 (7.1)	2 (13.3)
**Marital status**						
Single/widowed/divorced/separated	3 (27.3)	20 (58.8)	14 (58.3)	53 (59.6)	8 (57.1)	7 (46.7)
Married	8 (72.7)	14 (41.2)	10 (41.7)	36 (40.4)	6 (42.9)	8 (53.3)
**Religion**						
Buddhism	8 (72.7)	22 (64.7)	10 (41.7)	54 (60.7)	9 (64.3)	7 (46.7)
Islam	2 (18.2)	8 (23.5)	10 (41.7)	28 (31.5)	4 (28.6)	4 (26.7)
Christianity/Other	1 (9.1)	4 (11.8)	4 (16.7)	7 (7.9)	1 (7.1)	4 (26.7)
**Level of education**						
Secondary or below	5 (45.5)	10 (30.3)	6 (25.0)	15 (16.9)	4 (28.6)	4 (26.7)
High school/diploma	4 (36.4)	13 (39.4)	6 (25.0)	28 (31.5)	4 (28.6)	9 (60.0)
Bachelor’s degree or higher	2 (18.2)	10 (30.3)	12 (50.0)	46 (51.7)	6 (42.9)	2 (13.3)
**Occupation**						
Government officer/state enterprise employee/company employee	3 (27.3)	7 (20.6)	6 (25.0)	22 (24.7)	3 (21.4)	3 (20.0)
Self-employed/merchant/personal business owner/agriculture	6 (54.5)	18 (52.9)	11 (45.8)	37 (41.6)	6 (42.9)	7 (46.7)
Student	0 (0.0)	4 (11.8)	1 (4.2)	15 (16.9)	2 (14.3)	1 (6.7)
Unemployed	2 (18.2)	5 (14.7)	6 (25.0)	15 (16.9)	3 (21.4)	4 (26.7)
**Diagnosis**						
Depression	5 (45.5)	15 (44.1)	11 (45.8)	36 (40.4)	6 (42.9)	5 (33.3)
Anxiety/panic	4 (36.4)	8 (23.5)	3 (12.5)	23 (25.8)	1 (7.1)	3 (20.0)
Bipolar/schizophrenia	2 (18.2)	5 (14.7)	5 (20.8)	15 (16.9)	4 (28.6)	6 (40.0)
Other	0 (0.0)	6 (17.6)	5 (20.8)	15 (16.9)	3 (21.4)	1 (6.7)
**Presence of LGBTQ + close relatives/friends**						
Yes	3 (27.3)	13 (38.2)	6 (25.0)	29 (32.6)	3 (21.4)	3 (20.0)
No	8 (72.7)	21 (61.8)	18 (75.0)	60 (67.4)	11 (78.6)	12 (80.0)
**Prior experience of medical examination by an LGBTQ + physician**						
Yes	2 (18.2)	4 (11.8)	3 (12.5)	20 (22.5)	1 (7.1)	2 (13.3)
No	9 (81.8)	30 (88.2)	21 (87.5)	69 (77.5)	13 (92.9)	13 (86.7)
**Patient-doctor relationship**						
Poor	0 (0.0)	0 (0.0)	0 (0.0)	2 (2.2)	0 (0.0)	0 (0.0)
Moderate	1 (9.1)	12 (35.3)	7 (29.2)	25 (28.1)	2 (14.3)	4 (26.7)
Good	10 (90.9)	22 (64.7)	17 (70.8)	62 (69.7)	12 (85.7)	11 (73.3)
**Median attitude score**	63	66	62	70	63	62
Interquartile range (IQR)	59.0–66.5	59.3–78.8	57.0–68.3	62.0–80.0	56.3–66.0	54.0–66.0

### Associations between demographic characteristics and perceived attitudes toward LGBTQ + physicians

The selection process began with a univariate analysis of each variable. Any variable having a significant univariate test (*p*-value < 0.2) was selected as a candidate for the multivariate analysis. The final model from linear regression analyses revealed that age, religion, level of education, matched sex and gender, presence of LGBTQ + close relatives/friends, and patient-doctor relationship were significantly associated with the perceived attitudes toward LGBTQ + physicians. Our result showed that the older age groups reported lower scores of perceived attitudes towards LGBTQ + physicians compared to the younger age groups. Similarly, Muslims reported lower attitude scores than Buddhists (adjusted coefficient: -9.31, 95% CI = -11.48, -7.15). In addition, those reporting an absence of LGBTQ + close relatives/friends had lower perceived attitude scores (adjusted coefficients: -7.07, 95% CI = -9.03, -5.01). Meanwhile, a higher level of education was positively associated with the score of perceived attitudes toward LGBTQ + physicians (adjusted coefficient: 6.63, 95% CI = 3.95, 9.30 for Bachelor’s degree or higher level of education, and 3.0, 95% CI = 0.18, 5.82 for high school/diploma level of education). Moreover, the self-report of a sex-gender mismatch was positively associated with the score of perceived attitudes toward LGBTQ + physicians (adjusted coefficient: 10.42, 95% CI = 5.39, 15.45) (Table 5).

**Table 5:** Linear regression models of attitude scores toward LGBTQ + physicians (*N* = 542)

**Variables**	**Crude coefficient** (95% CI)	**Adjusted coefficient** (95% CI)	***P*-value** (F-test)
**Matched sex and gender**			< 0.001
Male	Reference	Reference	
Female	6.94 (4.54, 9.34)	3.66 (1.56, 5.75)	
Mismatch	17.93 (11.98, 23.89)	10.42 (5.39, 15.45)	
**Age group** (years)			< 0.001
18–25	Reference	Reference	
26–40	-7.77 (-10.52, -5.01)	-5.77 (-8.15, -3.40)	
41–60	-11.34 (-14.34, -8.33)	-9.12 (-11.91, -6.34)	
> 60	-12.38 (-16.97, -7.78)	-9.74 (-13.84, -5.64)	
**Religion**			< 0.001
Buddhism	Reference	Reference	
Islam	-7.71 (-10.26, -5.17)	-9.31 (-11.48, -7.15)	
Christianity/other	-2.65 (-6.85, 1.55)	-1.62 (-5.05, 1.82)	
**Level of education**			< 0.001
Secondary school and below	Reference	Reference	
High school/diploma	5.31 (2.02, 8.60)	3.00 (0.18, 5.82)	
Bachelor’s degree and above	10.72 (7.62, 13.81)	6.63 (3.95, 9.30)	
**Diagnosis**			0.052
Depression	Reference	Reference	
Anxiety/GAD	-5.77 (-8.52, -3.03)	-0.38 (-2.73, 1.97)	
Bipolar/schizophrenia	-7.45 (-11.16, -3.74)	-4.08 (-7.18, -0.99)	
Other	-3.60 (-7.12, -0.07)	0.37 (-2.62, 3.35)	
**Presence of LGBTQ + close relatives/friends**			< 0.001
Yes	Reference	Reference	
No	-10.98 (-13.12, -8.83)	-7.07 (-9.03, -5.01)	
**Patient-doctor relationship**			< 0.001
Poor	Reference	Reference	
Moderate	0.60 (-11.61, 12.80)	-0.10 (-9.85, 9.66)	
Good	4.98 (-7.07, 17.03)	4.10 (-5.53, 13.74)	

Adjusted R-squared: 0.3868

## Discussion

To our knowledge, this is the first multihospital-based study investigating the perceived attitudes toward LGBTQ + physicians and their associating factors among individuals with psychiatric illnesses in Thailand. It found that, among its 542 participants, the score of the perceived attitudes toward LGBTQ + physicians was 75, which indicated a relatively positive attitude. Most participants recognized LGBTQ + physicians as normal, and that being an LGBTQ + physician was not immoral. In addition, LGBTQ + physicians constitute a viable part of society and should enjoy full acceptance by society. However, older patients, Islam, and lacked LGBTQ + close relatives/friends reported lower attitude scores toward LGBTQ + physicians. A higher level of educational attainment and a self-report of a sex-gender mismatch were positively associated with a higher score of perceived attitudes toward LGBTQ + physicians. When looking into the participants’ perceived attitudes toward LGBTQ + physicians versus their attitudes toward non-LGBTQ + physicians, it was clear that our study participants felt comfortable with LGBTQ + physicians during the processes of taking a history of general matters, physical examination of general body parts, and management of both medical and psychiatric conditions. However, they reported feeling comfortable during the physical examination of private parts, such as breasts and genitalia, by LGBTQ + physicians in a lower proportion.

Among our psychiatric study samples, the overall score of the perceived attitudes toward LGBTQ + physicians can be considered a high score and a fair reflection of the positive attitudes toward LGBTQ + physicians. This finding was similar to those of prior reports [19].

The finding reiterates that LGBTQ + perspectives are becoming less pathologic and/or less separated from those considered to be the societal norms than before [3]. However, there were differences from other Asian nations, such as Malaysia, that there remains a stigma against individuals with LGBTQ + preferences [20]. Therefore, the different results could be attributed to the nature of the sample, i.e., ethnicity, culture, and individuals with psychiatric illnesses may have different characteristics or attitudes compared to individuals with other illnesses or members of the general population. Moreover, nearly half of our participants reported having LGBTQ + close relatives/friends, and they had a previous experience of being medically examined by an LGBTQ + physician. Therefore, having direct prior experiences with LGBTQ + individuals, both positive and negative aspects, may have influenced perceived attitudes toward LGBTQ + physicians in this study. Additionally, the more positive view of society toward LGBTQ + people at present [21] may be a possible influence on the improvement of the views toward LGBTQ + physicians among individuals with psychiatric illnesses. Additionally, 21 participants (3.9%) reported a mismatch of their sex and gender. Among them, the median (IQR) score of the perceived attitudes toward LGBTQ + physicians was 94 (85, 95), which was significantly higher than the median score of participants for whom sex and gender were matched (*p* < 0.001). Therefore, it may be possible that these participants are LGBTQ + individuals, which may have influenced their high scores or reported positive attitudes toward LGBTQ + physicians.

Concerning the patient-physician relationship, the perception of the physician's identity [5] or physician's gender [22] on the part of patients is one of the main categories of barriers that exist between patients and physicians [5]. In particular, there were statistically significant differences in the care process of history taking and physical examination in the private organ [9, 10]. Although this study found that most participants reported a good doctor-patient relationship and positive perceived attitudes towards LGBTQ + physicians, 6.3% and 16.4% of our participants reported feeling uncomfortable during history taking involving private matters and physical examinations involving sexual organs. Furthermore, a difference in such attitudes was detected between age groups. The findings were similar to those of prior reports [9, 10]. This may reflect that even though they identify high scores of positive attitudes towards LGBTQ + physicians, in some age groups, and in situations where privacy is compromised, feelings of mistrust may arise, and these findings may also be found in individuals with general medical conditions. A potential explanation for these findings may be the fact that the history taking covering private matters, e.g., sexual relationships, history of psychiatric illness, etc., or the physical examination of private body parts requires a deeper relationship and/or a higher degree of trust than the history taking concerning less private matters or the physical examination of body parts not considered private. Moreover, another factor in this regard is the fact that it is more common for individuals with psychiatric illnesses may have a deficit in fulfilling relationships with others or have problems with mistrust of others than it is for other patients [11]. Additionally, this study found that having prior experience of medical examination by an LGBTQ + physician did not lead to different attitudes toward LGBTQ + physicians than having no such experience. It is also possible that, in addition to attitudes toward LGBTQ + physicians, there may be other psychological factors that are associated with the feelings of discomfort experienced by individuals with psychiatric illnesses when receiving medical care from LGBTQ + physicians.

This topic requires further in-depth study.

Additionally, our older age and Muslim participants reported lower scores of perceived attitudes toward LGBTQ + physicians, while those with a higher level of education reported higher scores. When looking at prior studies conducted in Southern Thailand, they found that individuals with general medical conditions of certain age groups, gender, and religion had low rates of positive views regarding LGBT physicians [9, 10]; the findings of the current study go in the same direction as those results. This may be due to religious beliefs and perspectives on accepting different things in that age group that have not changed from the past. Consequently, to ensure an optimal quality of care delivered by LGBTQ + physicians for individuals irrespective of whether they have general medical conditions or psychiatric illnesses, if the patient belongs to a certain age group, has a certain level of education, and adheres to a particular religion, LGBTQ + physicians should be extra attentive and try their hardest to act in a manner that is conducive toward building trust with them.

This study has a few noteworthy strengths and limitations. To our knowledge, this is the first study investigating the perceived attitudes toward LGBTQ + physicians among individuals with psychiatric illnesses in Thailand. It provides a current reflection of the psychiatric individuals' perceived attitudes toward LGBTQ + physicians, especially as it regards the Southern Thai context. However, this study provides results with a limited scope of interpretation due to its cross-sectional design as well as utilization of self-administered questionnaires, which suffer from the inherent possibility of misunderstandings on the part of respondents regarding the intended meaning of the questions. Nevertheless, to minimize this, questionnaires with good reliability were utilized (good Cronbach’s alpha coefficient values). Another drawback was the fact that our data were quantitative, which may not provide in-depth qualitative answers. Furthermore, our participant's mental capacity was selected under the supervision of an outpatient psychiatric nurse who was not their psychiatrist. Therefore, there might be selection bias. Additionally, a sample group was limited to patients from Southern Thailand. Even though we tried to collect data from four hospitals with different characteristics, which serve areas with diverse populations in terms of religion and culture as well as patients with all types of psychiatric illnesses, most of our participants were patients with MDD and GAD; this means that not all psychiatric illnesses were fairly represented. In addition, this dataset might not fairly represent Thai individuals with psychiatric illnesses countrywide due to the differences in the ethnic, religious, and cultural make-up that exist between the population of the Southern Region and those of the other regions of Thailand. Therefore, it is recommended that future studies include patients with a greater variety of psychiatric illnesses from all regions of Thailand. In other words, a comprehensive multi-region study should be conducted. Moreover, such a study should utilize different study instruments and a more qualitative design as well as employ a more in-depth approach.

## Conclusion

Most participants reported positive perceived attitudes toward LGBTQ + physicians. However, participants belonging to some age groups or religions showed lower attitude perception scores and reported feeling uncomfortable receiving care from LGBTQ + physicians. On the one hand, LGBTQ + physicians in Southern Thailand have cause for concern regarding this finding, on the other hand, more attention and effort needs to be dedicated toward finding ways to promote positive attitudes toward LGBTQ + people in general and physicians in particular among these groups of people.

## Data Availability

No datasets were generated or analysed during the current study.

## Abbreviations

LGBTQ +: Lesbian, gay, bisexual, transgender, queer or questioning, and more
ATTI: Attitudes toward transgendered individuals scale
PDRQ: Patient-doctor relationship questionnaire

## References

[1] Bouton RA, Gallaher PE, Garlinghouse PA, Leal T, Rosenstein LD, Young RK (1989). Demographic variables associated with fear of AIDS and homophobia. J Appl Soc Psychol.

[2] Hunt L, Vennat M, Waters JH (2018). Health and wellness for LGBTQ. Adv Pediatr.

[3] Herek GM (2007). Confronting sexual stigma and prejudice: Theory and practice. Aust J Soc Issues.

[4] Kitts RL (2010). Barriers to optimal care between physicians and lesbian, gay, bisexual, transgender, and questioning adolescent patients. J Homosex.

[5] Keshavarzi MH, Safaie S, Faghihi SAA, Zare S (2022). Barriers of physician-patient relationships in professionalism: A qualitative study. J Adv Med Educ Prof.

[6] Hahn H, van SeagerDyk I, Ahn WY (2019). Attitudes toward gay men and lesbian women moderate heterosexual adults' subjective stress response to witnessing homonegativity. Front Psychol..

[7] Meyer IH (2003). Prejudice, social stress, and mental health in lesbian, gay, and bisexual populations: conceptual issues and research evidence. Psychol Bull.

[8] Aleshire ME, Ashford K, Fallin-Bennett A, Hatcher J (2019). Primary care providers' attitudes related to LGBTQ+ people: a narrative literature review. Health Promot Pract.

[9] Pitanupong J, Sam-angsri N (2011). Patient’s attitudes towards overtly effeminate male or overtly masculine female doctors. J Psychiatr Assoc Thailand.

[10] Pitanupong J, Wittayanont A (2012). Overtly effeminate male or overtly masculine female doctors from patients’ and their relatives’ point of view. Songkla Med J.

[11] Block VJ, Haller E, Villanueva J, Meyer A, Benoy C, Walter M (2022). Meaningful relationships in community and clinical samples: their importance for mental health. Front Psychol.

[12] Lee SR, Kim MA, Choi MN, Park S, Cho J, Lee C (2021). Attitudes toward transgender people among medical students in South Korea. Sex Med.

[13] Ngamake ST, Walch SE, Raveepatarakul J (2013). Validation of the attitudes toward transgendered individuals scale in Thailand. Int J Transgend.

[14] Pitanupong J, Sangkool J, Jatchavala C (2018). Outpatients’ preference and attitudes toward the Thai’s physician attire: A cross-sectional study. Songkla Med J.

[15] Pitanupong J, Sammathit J (2023). Knowledge and attitudes on medication adherence and residual symptoms in individuals with depression: a survey at a University Hospital. BMC Psychiatry.

[16] Alomran AM, Almubarak DA, Alrashed BA, Khan AS (2020). Psychological insulin resistance among type 2 diabetic patients attending primary healthcare centers, Al-Ahsa. Saudi Arabia J Family Community Med.

[17] Van der Feltz-Cornelis CM, Van Oppen P, Van Marwijk HWJ, De Beurs E, Van Dyck R (2004). A patient-doctor relationship questionnaire (PDRQ-9) in primary care: development and psychometric evaluation. Gen Hosp Psychiatry.

[18] Arafat SM (2016). Psychometric validation of the Bangla version of the patient-doctor relationship questionnaire. Psychiatry J.

[19] Walch SE, Ngamake ST, Francisco J, Stitt RL, Shingler KA (2012). The attitudes toward transgendered individuals scale: psychometric properties. Arch Sex Behav.

[20] Juhari JA, Gill JS, Francis B (2022). Coping Strategies and Mental Disorders among the LGBT+Community in Malaysia. Healthcare.

[21] Fetner TUS (2016). attitudes toward lesbian and gay people are better than ever. Contexts.

[22] Douglas SL, De Souza LR, Yudin MH (2017). Identification of patient-perceived barriers to communication between patients and physicians. Fam Med Med Sci Res.

